# Diagnosis of peripheral neuropathy

**DOI:** 10.1186/s42466-020-00064-2

**Published:** 2020-07-15

**Authors:** Helmar C. Lehmann, Gilbert Wunderlich, Gereon R. Fink, Claudia Sommer

**Affiliations:** 1 Department of Neurology, Faculty of Medicine and University Hospital of Cologne, Kerpener Straße 62, D-50937 Köln, Germany; 2 Center for Rare Diseases, Faculty of Medicine and University Hospital of Cologne, Köln, Germany; 3 Institute of Neuroscience and Medicine (INM-3), Research Centre Juelich, Jülich, Germany; 4 Department of Neurology, University of Würzburg, Würzburg, Germany

**Keywords:** Peripheral neuropathy, Diagnosis, EMG, Nerve conduction studies, Hereditary amyloid transthyretin (ATTRv) amyloidosis, CIDP, Diabetic, Ultrasound

## Abstract

**Introduction:**

Peripheral neuropathy represents a spectrum of diseases with different etiologies. The most common causes are diabetes, exposure to toxic substances including alcohol and chemotherapeutics, immune-mediated conditions, and gene mutations. A thorough workup including clinical history and examination, nerve conduction studies, and comprehensive laboratory tests is warranted to identify treatable causes.

**First steps:**

The variability of symptoms allows distinguishing characteristic clinical phenotypes of peripheral neuropathy that should be recognized in order to stratify the diagnostic workup accordingly. Nerve conduction studies are essential to determine the phenotype (axonal versus demyelinating) and severity. Laboratory tests, including genetic testing, CSF examination, nerve imaging, and nerve biopsy, represent additional clinical tests that can be useful in specific clinical scenarios.

**Comments:**

We propose a flow chart based on five common basic clinical patterns of peripheral neuropathy. Based on these five clinical phenotypes, we suggest differential diagnostic pathways in order to establish the underlying cause.

**Conclusions:**

The recognition of characteristic clinical phenotypes combined with nerve conduction studies allows pursuing subsequent diagnostic pathways that incorporate nerve conduction studies and additional diagnostic tests. This two-tiered approach promises higher yield and better cost-effectiveness in the diagnostic workup in patients with peripheral neuropathy.

## Introduction

Peripheral neuropathies are among the most common neurological diseases with an incidence of 77/100,000 inhabitants per year and a prevalence of 1–12% in all age groups and up to 30% in older people [1–3]. In the USA, it is estimated that patients with idiopathic neuropathies outnumber patients with Alzheimer’s disease up to threefold [4].

The diagnosis of peripheral neuropathy necessitates a thorough workup of possible etiologies in order to identify treatable causes of this disease spectrum as early as possible. For instance, almost every 10th patient suffers from a polyneuropathy of autoimmune origin [1], which is amenable to causal (immunosuppressive or immunomodulatory) therapies and, therefore, must not be overlooked. Recently, even hereditary neuropathies have entered the “era of treatment in neurology”, with the approval for transthyretin stabilizing agents (tafamidis), RNA interference molecules (patisiran) and antisense oligonucleotids (inotersen) in hereditary transthyretin amyloidosis (ATTR_v_).

Hospital data-based epidemiological studies provide (often differing) lists of most frequent causes of peripheral neuropathy in Western countries (Table 1). Unfortunately, epidemiological data about causes of peripheral neuropathies in other geographical regions such as Asia or South America are sparse. Importantly, patients may occasionally suffer from more than one disease causing their peripheral neuropathy. Clinically relevant co-occurrences are, for example, diabetes mellitus and chronic inflammatory demyelinating polyradiculoneuropathy (CIDP), HIV infection and CIDP, or diabetes mellitus and chronic alcohol misuse.

**Table 1:** Causes of peripheral neuropathy according to studies in Norway and the Netherlands

	Norway [5]	Netherlands [1]	USA [6]
Number of patients:	226	743	231
Idiopathic axonal^b^	28%	26%	12%
Diabetic	18%	32%	46%
Toxic (alcohol, drugs chemotherapy etc.)	10%	14%	13%
Inflammatory / Immune-mediated	16%	9%	8%
Hereditary	14%	5%	7%
Vasculitic, amyloid neuropathy, sarcoid, connective tissue disease	^a^	5%	1%
Uremic, thyroid dysfunction	^a^	4%	3%
Vitamin B12 deficiency	4%	3%	1%
Others (i.e. idiopathic small fiber neuropathy^b^)	10%	2%	

^a^ = not classified, ^b^ = axonal in the study from the Netherlands

**Fig. 1:**
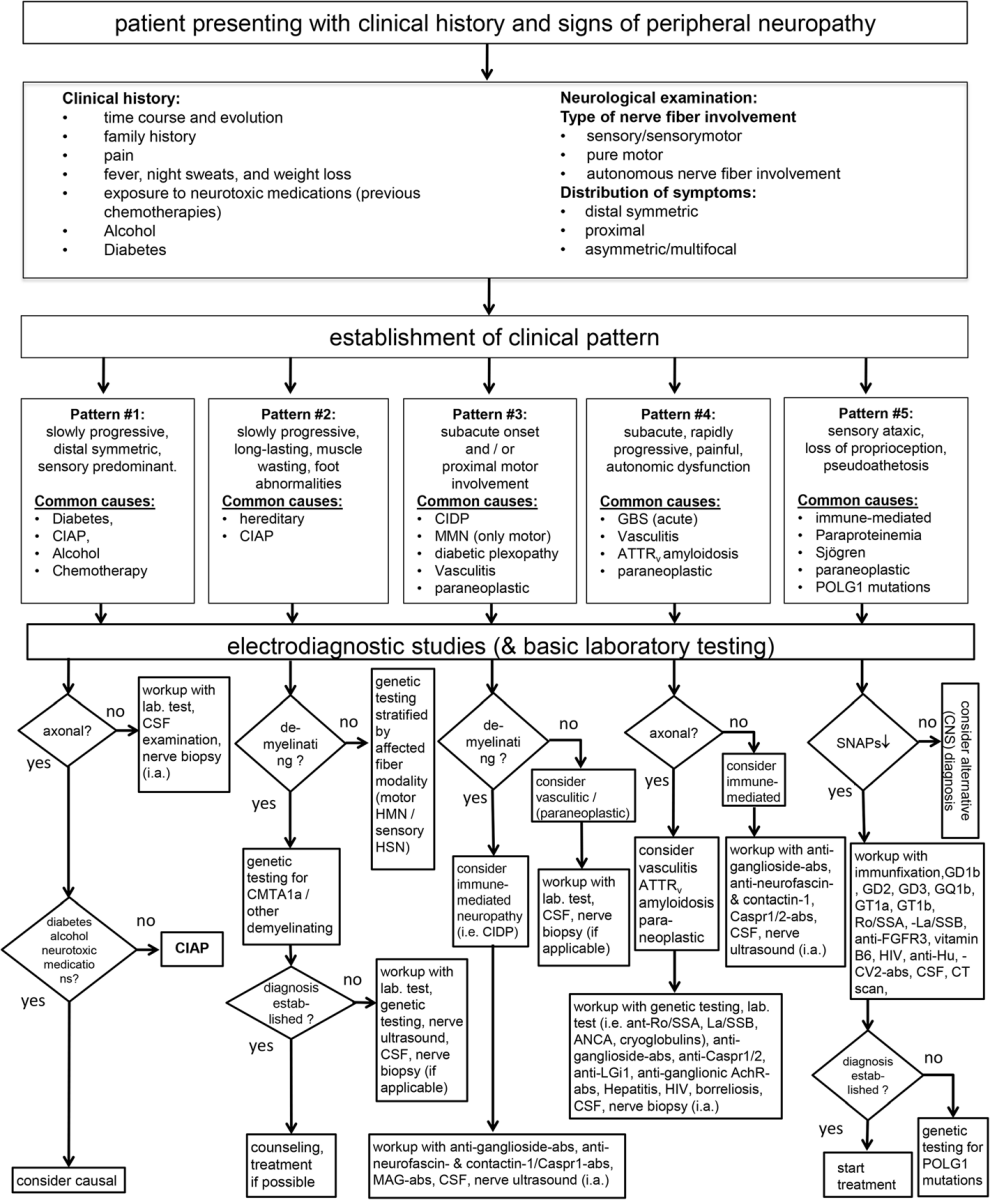
Flow chart of a diagnostic algorithm for the workup of patients with peripheral neuropathy. According to the established clinical patterns, based on clinical history and examination, diagnostic procedures can be stratified. Abs = antibodies, ATTRv = hereditary transthyretin amyloidosis, CIAP = chronic idiopathic axonal polyneuropathy, CSF = cerebrospinal fluid, i.a.= if applicable, SNAP = sensory nerve action potential

Evidence-based guidelines and diagnostic algorithms for the diagnosis of peripheral neuropathy have been published for specific neuropathic phenotypes such as distal symmetric peripheral neuropathy [7–9], small fiber neuropathy, or inflammatory neuropathies. However, they are only applicable for specific neuropathic conditions or when a specific diagnosis is already suspected on clinical grounds. By focusing on the initial steps of the diagnostic workup, this standard operating procedure provides a practical guideline including clinical and additional diagnostic parameters that help to identify the underlying cause of peripheral neuropathy. Figure 1 provides a flow chart of this diagnostic workup.

### First steps

Recognizing specific clinical patterns is essential to stratify the diagnostic workup in a patient who presents with signs and symptoms of peripheral neuropathy. This workup should include a detailed history and a thorough clinical examination. In our flow chart, we propose five different clinical patterns:

- #1. Slowly progressive, distal symmetric, predominantly sensory neuropathy: This most common peripheral neuropathy subtype is often caused by a metabolic condition (diabetes), chronic alcohol consumption, or neurotoxic drugs (chemotherapy). These patients only need limited diagnostic testing unless atypical neuropathy features are present. Exclusion of these causes may lead to the diagnosis of chronic idiopathic axonal neuropathy (CIAP), which usually has a benign course.
- #2. Slowly progressive, long-standing neuropathy with muscle wasting and foot abnormalities: Motor predominant, onset in child-, or adulthood, these patients may be less frequent compared to the other subtypes. Diagnostic workup should be prioritized towards genetic testing.
- #3. Neuropathy with subacute onset and/or proximal involvement: These patients present with clinical features suggestive of an acquired immune-mediated condition. Extensive diagnostic workup, including antibody testing, etc. may be required.
- #4. Neuropathy with subacute or rapidly progressive disease course, multifocal symptoms, neuropathic pain, and autonomic dysfunction: Potentially caused by vasculitis, amyloidosis, or as paraneoplastic syndrome. Patients with this subtype should undergo detailed diagnostic workup.
- #5. Sensory ataxic neuropathy: Clinical correlates of sensory neuronopathy or Denny-Brown’s syndrome. Patients present with loss of proprioception and vibration sense and may display pseudoathetosis, with relative preservation of muscle strength. Underlying causes that should be explored include autoimmune disorders (i.e., Sjögren), paraneoplastic syndromes, and mitochondrial disorders.

These five subtypes should neither be taken as exclusive nor absolute since overlap of these patterns is not uncommon. For instance, some patients with a hereditary neuropathy (i.e., ATTRv amyloidosis) present with a rapidly progressive disease course, and are often misdiagnosed as CIDP. On the other hand, also CIDP patients occasionally present with a slowly progressive disease course.

### Clinical history

**Fig. 2:**
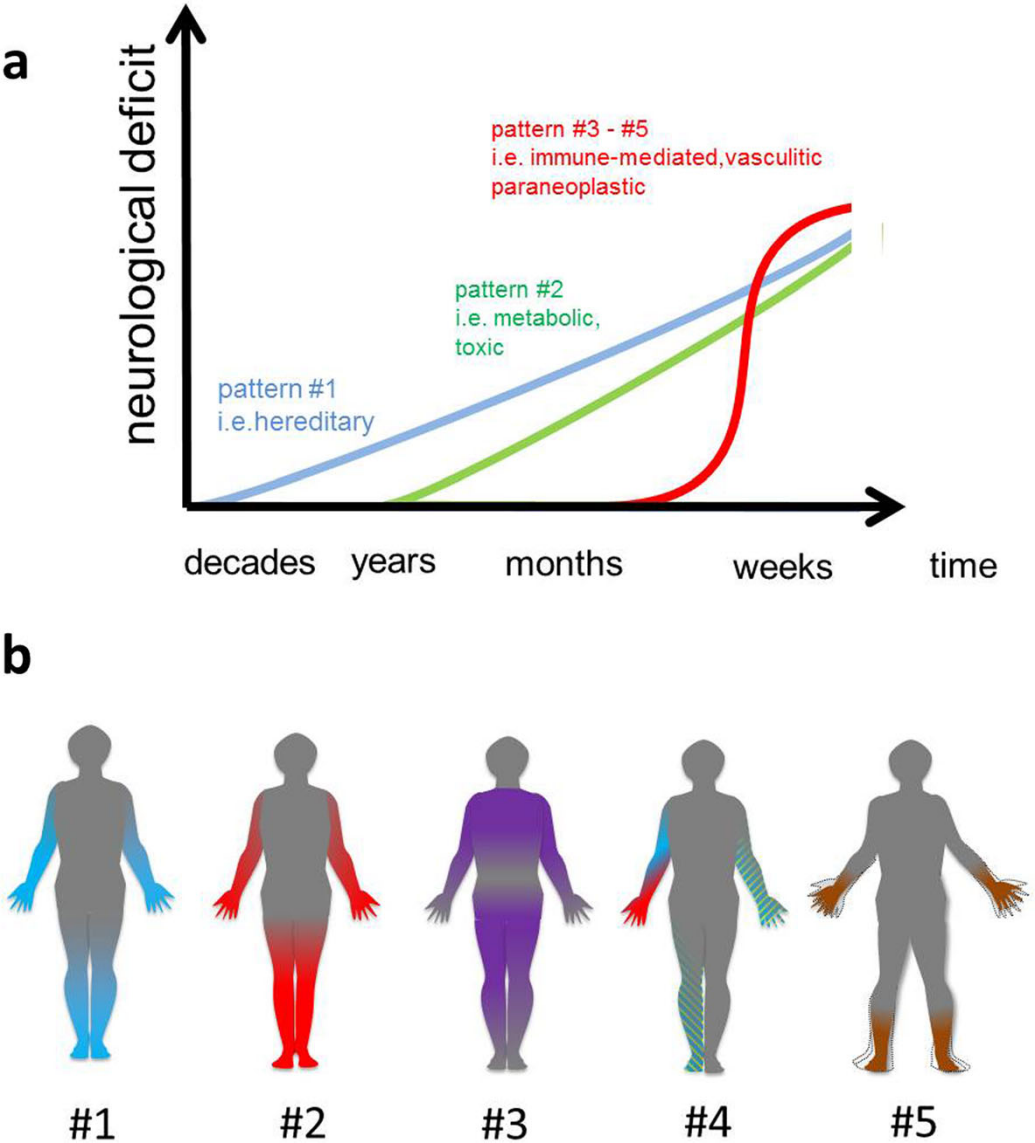
**a** Disease onset and temporal evolution characteristics of distinguishable clinical patterns and different causes of peripheral neuropathy. **b** Clinical patterns of polyneuropathy: Sensory deficits are drawn in blue, motor deficits are drawn in red, and sensorimotor in magenta color. Painful and / or autonomous dysfunction is colored with green lines. Loss of proprioception is colored in brown. Pattern #1 is a distal symmetric predominantly sensory neuropathy, #2 a motor neuropathy with muscle wasting and foot abnormalities; pattern #3 is characterized by proximal involvement of sensory and motor nerve fibers, pattern #4 presents wih multifocal symptoms, neuropathic pain, and autonomic dysfunction. Pattern #5: is a sensory ataxic neuropathy

Clinical history and presentation can provide valuable diagnostic hints toward an underlying cause of a peripheral neuropathy. A careful analysis of disease onset and its temporal evolution may indicate or exclude different forms of peripheral neuropathy. Most peripheral neuropathies are slowly progressive chronic diseases (clinical pattern #1). Neuropathic symptoms that slowly develop over decades, as can be observed in the clinical pattern #2, may be suspicious for a hereditary neuropathy, particularly when associated with prominent wasting and skeletal or foot deformities. (Sub) acute onset and evolution is characteristic for a clinical pattern #3 to #5 and may indicate inflammatory neuropathies, including the Guillain-Barré syndrome (GBS), chronic inflammatory demyelinating polyradiculoneuropathy (CIDP), vasculitis, paraneoplastic neuropathy, and diabetic lumbosacral radiculoplexus neuropathy [10] (Fig. 2a).

History-taking in patients with peripheral neuropathy should always include asking about fever, night sweats, weight loss (indicating hematological/oncological or chronic infectious disorder), exposure to neurotoxins (alcohol, previous chemotherapies, lead mercury, arsenic, and thallium), and diabetes. Besides, obtaining a careful family history can pave the way towards a diagnosis of inherited neuropathy. Particular symptoms and physical characteristics that should be questioned are claw hands, wasting of muscles, plantar foot ulcers, foot abnormalities. Even the examination of relatives with symptoms suggestive of inherited neuropathy should be considered.

### Neurological examination

Assessing the degree of involvement of different fiber modalities (motor, sensorimotor, sensory, autonomic nerve fibers), and the distribution of symptoms may further help to assign the patient to a particular clinical pattern (Fig. 2b).

#### Type of nerve fiber involvement

Most peripheral neuropathies are sensory or sensorimotor neuropathies. Pure or predominant motor signs qualify for clinical pattern #2 and #3 and occur in certain hereditary neuropathies or multifocal motor neuropathy, an immune-mediated neuropathy responsive to intravenous immunoglobulin treatment. Non-neuropathic conditions mimicking neuropathies (e.g., distal myopathies, amyotrophic lateral sclerosis, or spinal muscular atrophy) should be considered in patients with a lack of sensory involvement. A particular, though rare symptom complex constitutes early-onset ataxia and predominant loss of proprioception, which is a characteristic hallmark of sensory ganglionopathy / neuronopathy (clinical pattern #5).

Autonomic dysfunction can occur throughout all clinical patterns (but is often seen in clinical pattern #4) and may indicate diabetic neuropathy, wild type or ATTR_v_ amyloidosis, vincristine-induced neuropathy, or GBS [11]. The patient may fail to report (and sometimes even to recognize) symptoms of autonomic dysfunction. Accordingly, history taking should include symptoms of autonomic dysfunction, e.g., orthostatic intolerance, anhidrosis, dry eyes, dry mouth, constipation or diarrhea, impotence, tachycardia following sitting or standing, and hair loss in the distal legs [12].

#### Distribution of symptoms

Most neuropathies are length-dependent with a distal symmetric distribution of sensorimotor and/or autonomous neurological deficits. This distribution of symptoms is usually seen in clinical pattern #1 and #2. This becomes obvious when tendon reflexes are examined: ankle reflexes are usually absent, while more proximal reflexes can still be elicited. Sensory symptoms (e.g., hypesthesia) have a stocking and glove distribution pattern and may ascend proximally throughout the disease. Weakness and atrophy are most prominent in foot extensor muscles resulting in foot drop, or even only in toe flexors. It may indicate long-lasting neuropathy (i.e., clinical pattern #2). Prominent proximal weakness is characteristic for clinical pattern #3 and suggests an involvement of nerve roots or length-independent pathogenesis, which can be found in immune-mediated neuropathies or diabetic lumbosacral radiculoplexus neuropathy. Asymmetric neuropathies (multiplex mononeuritis) typically present with multifocal, often “patchy” symptoms and can be found in vasculitis and CIDP variants [13]. Cranial nerve involvement is only occasionally seen in polyneuropathy and may, therefore, be of diagnostic value. Neuropathies with cranial nerve involvement include diabetes mellitus (often monofocal), GBS, Lyme disease, sarcoidosis, diphtheria, or botulism. The latter can even be excluded on clinical grounds when cranial nerves are spared. Trigeminal nerve involvement is occasionally seen in paraneoplastic ganglionopathy (clinical pattern #5) [14].

### Electrodiagnostic studies

Nerve conduction studies (NCS) and needle electromyography (EMG) are carried out to

- confirm the clinical diagnosis of peripheral neuropathy
- exclude neuropathy mimics (i.e., radiculopathy, distal myopathy)
- reveal subclinical involvement of clinically unaffected nerves and fiber modalities
- assess the primary mechanism of damage (axonal vs. demyelinating), and
- determine disease severity.

After assigning the patient to a typical clinical pattern, the assessment of primarily axonal versus demyelinating nerve damage by NCS/EMG is a critical next step for further differential diagnosis. Most neuropathies are axonal, recognizable by reduced compound muscle action potentials (CMAP) in motor nerves, reduced sensory nerve action potentials (SNAP), and normal or slightly reduced nerve conduction velocities. These changes are typically found in patients with clinical pattern #1. The less frequently occuring demyelinating neuropathies are characterized by increased distal motor latencies, a significant slowing of nerve conduction velocities, conduction blocks, temporally dispersed potentials, and absent or delayed late responses (e.g., F-waves).

Clinical pattern #2 encompasses axonal and demyelinating neuropathies. Further assignment to one of these two forms of injury is essential for subsequent stratification of genetic testing. However, in long-lasting neuropathies, distinguishing these two fundamentally different injury patterns is sometimes problematic, since also demyelinating neuropathies invariably go along with some (secondary) axonal degeneration. On the other hand, amplitude-dependent slowing of nerve conduction studies may lead to the false assumption of a primarily demyelinating disorder. Therefore, consented rules for diagnosing demyelination are usually very strict [15].

A demyelinating neuropathy in patients who present with symptoms summarized in clinical pattern #3 or #4 is highly suggestive for an immune-mediated neuropathy. These acquired demyelinating neuropathies often have a patchy distribution of demyelinating features with different nerve conduction velocities. In contrast, uniform demyelination is more suggestive of an inherited neuropathy, i.e., Charcot-Marie-Tooth (CMT) type 1(A) [16]. Patients with clinical pattern #4 may have either axonal or demyelinating injury in NCS/EMG, and axonal damage may suggest vasculitis or ATTRv amyloidosis. Most patients presenting with clinical pattern #5 demonstrate reduced (often absent SNAPs) with normal motor CMAPs.

### Laboratory testing

Necessary laboratory testing (particularly in clinical pattern #1 and #2) includes a complete blood count, erythrocyte sedimentation rate, comprehensive metabolic panel (blood glucose, HbA1c, renal function, liver function), thyroid function tests, vitamin B12, and serum protein immunofixation [7, 9]. In distal symmetric polyneuropathy, the highest diagnostic yield was achieved with screening for blood glucose (including oral glucose tolerance test) and serum protein immunofixation (approx. every 10th patient positive) [7]. Serum vitamin B12 and cobalamine metabolites (methylmalonic acid and homocysteine) are also recommended since the latter are elevated in an additional 5–10% of patients whose serum B12 levels are in the lower normal range [7].

Clinical pattern #3 requires more extensive laboratory testing, including anti-ganglioside antibodies GM1, GD1a, neurofascin (NF155, NF186), contactin-1, Caspr1, and anti-myelin associated glycoprotein (MAG) antibodies. In patients with clinical pattern #4, serological testing for vasculitis (ACE, antinuclear antigen profile, rheumatoid factor, ant-Ro/SSA, anti-La/SSB, anti- neutrophil cytoplasmic antigen antibody (ANCA) profile, cryoglobulins), for immune-mediated neuropathies (anti-ganglioside-antibodies, anti-Caspr1/2, anti-LGi1, anti-ganglionic acetylcholine receptor antibodies) and infectious serology (Hepatitis B, and C, HIV, borreliosis) are recommended. Clinical pattern#5 should result in testing for anti-ganglioside antibodies (above all GD1b, GD2, GD3, GQ1b, GT1a, GT1b), anti-Ro/SSA, anti-La/SSB, anti-FGFR3, vitamin B6 (intoxication), HIV, anti-Hu, anti-CV2 antibodies. Here, also genetic testing should be considered for POLG1 (DNA polymerase subunit gamma) mutations.

Additional laboratory testing is usually not required (particularly in clinical pattern #1). It is only useful when additional general symptoms are present, i.e., gastrointestinal disease (anti-gliadin, anti-transglutaminase-antibodies, vitamin B levels), history for intoxications (blood, urine, hair and nail analysis for heavy metals e.g. arsenic, lead, mercury, thallium), or porphyria (porphyrin analysis in blood, urine, and stool). However, the yield of these additional tests is meager.

### Examination of the cerebrospinal fluid

Examination of the cerebrospinal fluid (CSF) is usually not instructive in slowly progressive symmetric polyneuropathy seen in clinical pattern #1 and #2. CSF examination is warranted when an inflammatory, vasculitic, paraneoplastic, or infectious cause is suspected (clinical pattern #3 - #5). In immune-mediated neuropathies, albuminocytological dissociation is often found, whereas infectious causes result in CSF pleocytosis. Oligoclonal bands can be found in paraneoplastic neuropathy, borreliosis, sarcoidosis, M. Behçet, and other inflammatory conditions.

### Genetic testing

Genetic testing should be considered when clinical history or examination suggests a hereditary origin of the peripheral neuropathy (i.e., clinical pattern #2, #4, occasionally #5). Positive family history is the most apparent hint but may be absent in the case of de novo mutations, adopted individuals, or small families [17]. Symptoms that develop over decades, prominent wasting, and skeletal or foot deformities are clinical clues to a hereditary neuropathy (clinical pattern #2). Young age at onset is also suggestive for hereditary neuropathy. However, there are many examples of late-onset hereditary neuropathy, e.g., axonal CMT or ATTR_v_ amyloidosis.

Genetic testing can be further stratified according to the mode of inheritance, demyelinating versus axonal pattern, and affected nerve fiber modality [17]. For instance, in patients with positive family history and demyelinating neuropathy, 70% have a duplication of the PMP22 gene (CMT1A), whereas in patients with positive family history and axonal neuropathy, 33% have a mutation in MFN2 [7]. In general, about 90% of hereditary neuropathies are caused by either PMP22, MFN2, MPZ, and Cx32, respectively [16]. Testing for ATTR_v_ amyloidosis should be considered in patients presenting with the following red flags, i) origin from endemic regions (Portugal, Japan, Sweden), ii) rapid progressive, often painful peripheral neuropathy with prominent autonomic involvement (clinical pattern #4), and iii) systemic symptoms like cardiomyopathy or cachexia. Sensory ataxic neuropathy is occasionally caused by mitochondrial disease; thus genetic testing for POLG1 mutations should be considered in patients with clinical pattern #5.

### Nerve biopsy

Nerve biopsy as an invasive procedure should be considered in patients presenting with symptoms and signs suggestive of an inflammatory neuropathy. Mainly when a non-systemic vasculitic neuropathy is suspected, nerve biopsy is mandatory to confirm the diagnosis. In contrast, for demyelinating immune-mediated neuropathies, nerve biopsy is not required to fulfill the diagnostic criteria (i.e., CIDP) and should, therefore, only be done in case of diagnostic uncertainty. Based on the proposed clinical stratification, a biopsy might be only useful in patients with pattern#1 and #2, when atypical symptoms or additional tests suggest an acquired condition (i.e., vasculitis). In our experience, nerve biopsy has the highest yield in severe, rapidly progressive polyneuropathy (i.e., pattern #4), when vasculitis is suspected. Another indication may be treatment refractory inflammatory neuropathy to look for mimics. Usually, a sural nerve biopsy is performed. When the sural nerve is not affected, a fascicular biopsy from a different nerve can be performed guided by nerve imaging (see next section).

### Peripheral nerve imaging

Nerve ultrasound is another procedure that can be of diagnostic value in particular clinical scenarios [18], for example, when an immune-mediated neuropathy is suspected. Increased nerve cross-sectional areas can be found in most patients with immune-mediated neuropathy, especially in an asymmetrical distribution in arm nerves and roots. In contrast, a more uniform nerve enlargement is indicative of CMT1A. MRI can detect affection of proximal nerve segments that are not accessible by electrophysiology. Furthermore, MRI and nerve ultrasound can help to identify affected nerve segments, in order to target biopsy. At present, such techniques can only be recommended in selected cases and specialized centers.

### Other examinations

Depending on the clinical pattern (i.e., #4, #5), the test results, and the suspected underlying cause, it can sometimes become necessary to perform additional examinations, e.g., to exclude a malignancy by computed tomography of chest and abdomen or positron emission tomography.

## Conclusion

Early identification of an underlying cause of peripheral neuropathy is essential in order to initiate timely treatment, to prevent neurological sequelae, and to support self-management of affected patients. The development of specific treatment strategies for inherited neuropathies by RNA interference molecules and other approaches further emphasizes the value of establishing specific diagnoses in patients with peripheral neuropathy. On the other hand, even the diagnosis CIAP, which does not lead to a causal treatment, is of value since it allows counseling the patient about benign prognosis and prevents further useless and costly diagnostic tests. Thus the recognition of specific clinical phenotypes is a prerequisite pursuing differential, i.e., efficient diagnostic pathways that balance yield and cost-effectiveness. One should, however, keep in mind that polyneuropathy may be multifactorial. Nevertheless, the combination of medical history, clinical examination, NCS, and laboratory tests, reveals the etiology of the polyneuropathy in up to 4 of 5 patients [7]. Depending on the individual clinical scenario, diagnostic workup shown in the flow chart may need modification, for instance, if specific test results are positive or patients present with a clinical pattern not shown in the flow chart. Sometimes it may also become necessary to rule out other differential diagnoses by additional diagnostic tests.

## Data Availability

Not applicable.
